# Post-Transplant Immunosuppression: Regulation of the Efflux of Allospecific Effector T Cells from Lymphoid Tissues

**DOI:** 10.1371/journal.pone.0045548

**Published:** 2012-09-18

**Authors:** David J. Swan, John A. Kirby, Simi Ali

**Affiliations:** Applied Immunobiology and Transplantation Research Group, Institute of Cellular Medicine, Faculty of Medical Sciences, Newcastle University, Newcastle upon Tyne, United Kingdom; University of Pittsburgh, United States of America

## Abstract

A functional sphingosine-1-phosphate (S1P) receptor antagonist specifically inhibited the egress of activated allospecific T cells from draining popliteal lymph nodes in alloantigen-sensitised mice. The level of S1P receptor 1 (S1PR1) mRNA was similarly reduced 1 and 3 days after mitogenic activation of T cells. However, the response of these cells to the S1PR1-specific agonist SEW2871 was only reduced on the first day after T cell activation with normal receptor-mediated Akt-phosphorylation restored by day 3. Longitudinal analysis of CD69 expression showed that almost all T cells expressed this antigen on days 1 and 3 after activation. However, the absolute level of cell-surface expression of CD69 peaked on undivided T cells and was then halved by each of the first 3 cycles of mitosis. CD69-specific small interfering RNA (siRNA) reduced the maximal level of CD69 expression by undivided, mitogen-stimulated T cells. These cells retained their capacity to phosphorylate Akt in response to stimulation with SEW2871. These data show that S1P receptors are involved in controlling the egress of activated T cells from lymph nodes, and that S1PR1 function is regulated by the level of T cell surface CD69. They suggest a potential for augmentation of this process to deplete alloreactive effector cells after organ transplantation.

## Introduction

Immunosuppression produced by the inhibition of calcineurin has greatly enhanced the success of allogeneic organ transplantation. However, long-term administration of calcineurin inhibitors can cause a range of morbidities. For this reason there is a continuing pressure to develop new immunosuppressive drugs which function through different pathways. One novel strategy for the induction of immunosuppression involves inhibition of the efflux of activated T cells from the lymphoid tissues [1].

Under normal conditions, naïve T cells recirculate continually through blood and lymphatic tissues. Homeostatic chemokines, principally CCL19, CCL21 and CXCL12, drive entry into lymph nodes by promoting firm adhesion of T cells to high endothelial venules (HEV) followed by endothelial diapedesis [2]. These T cells remain in normal lymph nodes for between 6 and 24 hours before exiting via the cortical sinuses [3], [4]. This egress is driven by a positive concentration gradient of sphingosine-1-phosphate (S1P) from the lymph node to lymph, which stimulates the T cell-surface receptor S1PR1 [5], [6]. This model of T cell egress is supported by the action of the drug FTY720, which disrupts lymphocyte recirculation by inhibiting the normal response to S1P by binding S1PR1. This drug-receptor complex is then internalized and targeted for ubiquitination and degradation rather than recycling to the cell surface [7]–[9].

The drug FTY720, which is phosphorylated to FTY720-P *in vivo*, was found to prevent allograft rejection as effectively as calcineurin inhibitors [10], [11]. However, clinical trials were terminated as a consequence of adverse affects which, most significantly, included brachycardia. More specific analogues of FTY720 include AUY954 [1] and KRP-203 which is known to extend cardiac allograft survival in rodents [12]. Although new clinical trials are planned in transplantation, it is clear that our fundamental knowledge of regulation of the efflux of activated T cells from lymphoid tissues remains incomplete.

At the start of an adaptive immune response, only a small proportion of the T cells retained within a particular node is primed by TCR-specific interaction with antigen-presenting cells bearing immunogenic MHC antigen-peptide complexes. These T cells are triggered to proliferate and differentiate, generating effector and memory cells. Evidence suggests that the first effector T cells move into the circulation 3 days after the initial priming event [5]. This correlates with the period required for re-acquisition of the responsiveness to S1PR1 stimulation of activated T cells within the node [5].

It has been suggested that an activation-induced increase in the expression of S1PR1 and decrease in CCR7 are the most important factors resulting in the reacquisition of S1PR1 signalling and migration of effector T cells from lymph nodes [6]. However, this model does not include the potential involvement of CD69. CD69 is a type II TM protein of the C-type lectin family [13] and exists in its mature form in cells as a disulphide-linked dimer [14]. For a long time it was known simply as a marker that is upregulated onto the surface of T cells within hours of cell activation [15], [16]. More recently however, data from several studies have indicated an important role for the protein in the regulation of immune cell trafficking. Transgenic overexpression studies showed that CD69 inhibited thymocyte egress from the thymus [17], knockout of CD69 was shown to prevent lymph node shutdown [18] and adoptive transfer experiments with CD69-negative cells showed it was involved in relocation of memory T cells into the bone marrow compartment [19]. Immunoprecipitation and crosslinking-reporter assays showing direct interaction of CD69 with S1PR1 indicate a likely mechanism behind these phenomena. CD69 can form a complex with S1PR1 in the plasma membrane which leads to receptor internalization and degradation [18], [20].

The magnitude and dynamics of CD69 expression by T cells vary depending on the stimulus applied. For example, treatment for 1 h with type 1 IFNs induces low level expression of cell-surface CD69 [18], [21], whilst mitogenic T cell activation induces gene transcription leading to high level CD69 expression [16], [22]. These differences could be important for the control of S1PR1 signalling and regulation of the egress of T cells from lymph nodes.

The current study was designed to define roles played by the regulation of S1P-mediated signalling in controlling the egress of alloantigen-activated T cells from lymph nodes. An initial series of experiments was performed *in vivo* to demonstrate the potential of FTY720 to prevent the normal export of activated, alloantigen-specific T cells from reactive murine lymph nodes. Further experiments were performed *in vitro* to validate the relationship between the level of human T cell-surface CD69 expression and the response of S1PR1.S1PR1 function was tested using SEW2871 as an agonist, as the molecule has been shown to be highly selective for that S1P receptor. GTPγS binding assays using S1PR transfectants showed strong binding and signalling activity of SEW2871 through S1PR1, but a complete lack of activity of the ligand on S1PR2, 3,4 and 5 at a concentration of 10 µM [23].

**Figure 1:**
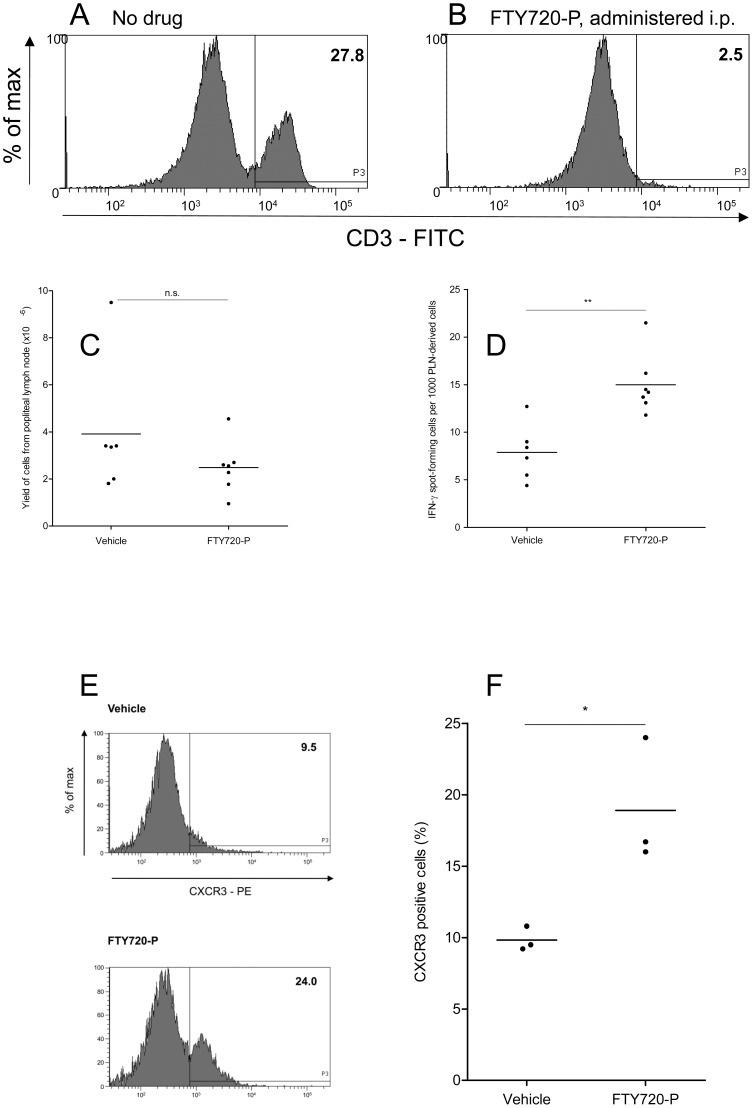
Activated T cells required S1P receptor signalling to exit lymph nodes. (A and B) C57BL/6 mice were injected i.p. with 100 µl of 100 µg/ml FTY720-P daily for two days, or left untreated. On day three, blood samples were taken from the tail vein and CD3-positive cells enumerated by flow cytometry. (C-F) Mice were injected with BALB/c splenocytes into the footpad on day zero and treated with FTY720-P or vehicle daily from days 2 to 5. On day six the popliteal lymph nodes were removed. (C) The nodes were disrupted and the total number of cells measured using a haemocytometer. (D) Popliteal node-derived cells were mixed with BALB/c splenocytes in IFN-γ cultured ELISPOT reactions. Data are presented as the number of IFN-γ spot-forming cells per 1000 popliteal node-derived cells. Points represent individual mice; 6 mice treated with vehicle, 7 with FTY720-P. Horizontal lines are means of groups. (E) Popliteal node cells were stained with an anti-mouse CXCR3 monoclonal antibody and analysed by flow cytometry. Flow histograms show representative staining of cells from one control mouse and one drug-treated mouse. Numbers are the percentage of lymph node-derived cells that were CXCR3-positive. (F) Graph showing CXCR3 expression by popliteal node cells derived from 3 control and 3 drug-treated mice. Points represent individual mice, horizontal lines are means of groups.

## Materials and Methods

### Reagents

FTY720 (S) phosphate (FTY720-P) was purchased from Cambridge Bioscience (Cambridge, UK). It was dissolved in ethanol at 1 mg/ml for storage at −20°C. On the day of use 1 mg/ml FTY720-P was diluted to 100 µg/ml in sterile water with 2% β-cyclodextrin (Sigma-Aldrich; Poole, UK) and then mixed thoroughly. SEW2871 was purchased from Enzo Life Sciences (Exeter, UK).

**Figure 2:**
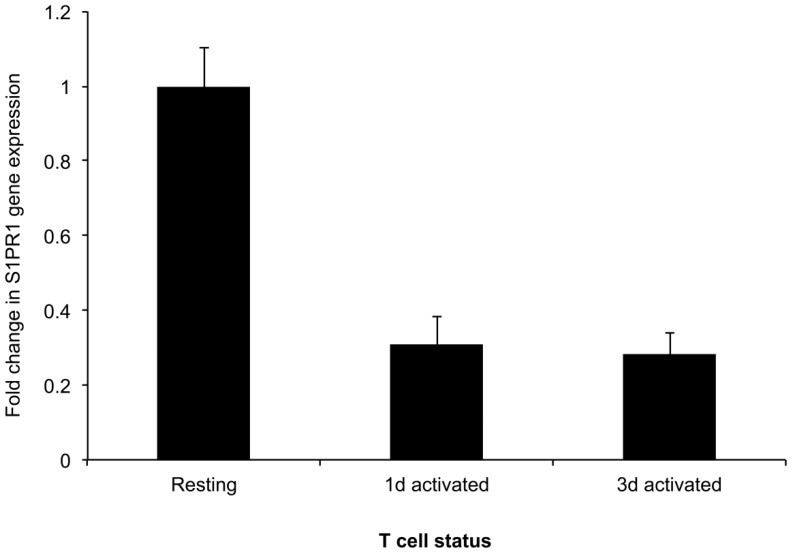
S1PR1 expression by resting and activated T cells. T cells were negatively selected from human peripheral blood. T cells were left at rest or stimulated for 1 or 3 days with mitogenic CD3/CD28 beads. The relative expression of mRNA encoding S1PR1 was determined in those cell populations by semi-quantitative RT-PCR. Data are representative of two independent experiments.

### Animals and Procedures

Female BALB/c (H2^d^) and C57BL/6 (H2^b^) mice (6–8 weeks old; Charles River, Margate, UK) were maintained under pathogen-free conditions. All procedures were performed in accordance with UK Home Office and EU Institutional Guidelines and within the parameters of current personal and project licences.

**Figure 3:**
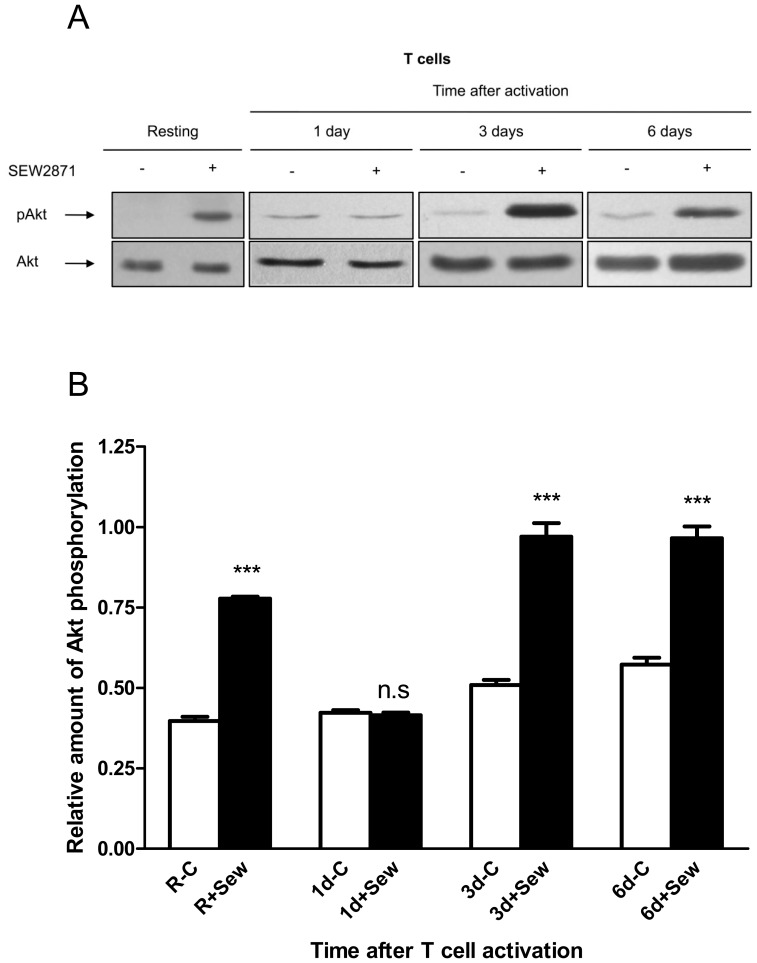
The capacity of S1PR1 to signal in T cells before and after cell activation. T cells were either left at rest or stimulated with CD3/CD28 beads for 1, 3 or 6 days. (A) The cells were then treated for 10 minutes with either 10 µM SEW2871 or vehicle before being lysed. Akt phosphorylated at residue serine 473 (pAkt) and total Akt were detected in cell lysates by western blotting. Blots are representative of those from three independent experiments. (B) Cell-based ELISA was used to quantify the relative amounts of pAkt and Akt in each cell population before and after cell stimulation with SEW2871. The relative amounts of pAkt in the samples were calculated by normalising the fluorescent signal value corresponding to pAkt to that of total Akt in each experimental well. Graph shows means ± s.e.m, n = 3 for each time point.

C57BL/6 mouse footpad injections (on day zero) were unilateral, subcutaneous, and comprised 5×10^6^ BALB/c splenocytes suspended in 25 µl RPMI 1640 medium (Sigma-Aldrich). Between days 2 and 5, some mice were injected daily, intraperitoneally, with 100 µl 100 µg/ml FTY720-P or an equal volume of drug vehicle. The mice were killed on day six and the popliteal lymph nodes draining the injected footpads removed. Cell suspensions were prepared from the nodes by pressing the tissue through 70 µm cell strainers (BD Biosciences; Oxford, UK) into RPMI 1640 medium. Popliteal lymph node-derived cells were washed twice with RPMI 1640 medium before further use.

**Figure 4:**
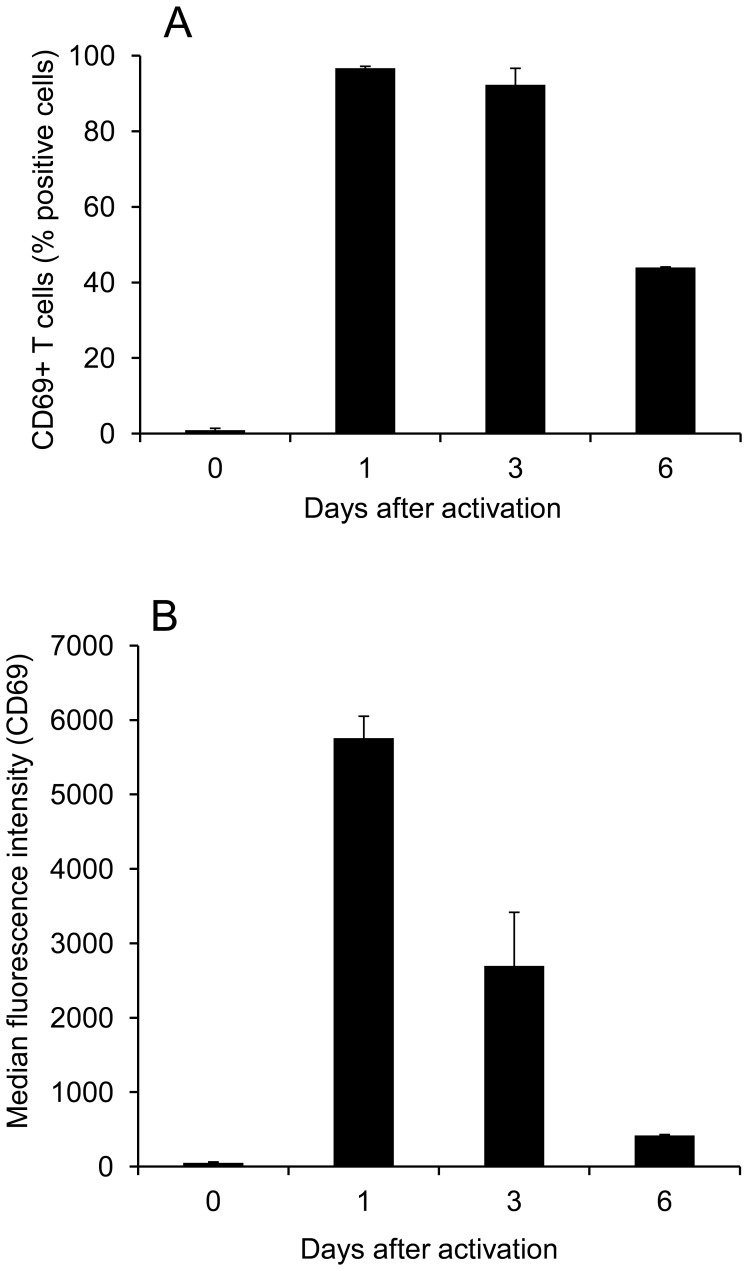
Dynamics of CD69 expression following T cell activation. Resting T cells were loaded with CFSE or not then either left at rest or stimulated with CD3/CD28 beads for 1, 3 or 6 days. The cells were then stained with a CD69 monoclonal antibody or with an isotype-matched control before flow cytometry. (A) Graph showing the percentage of T cells that were CD69-positive (excess specific staining over isotype) at each time-point. Data are representative of 2 or 3 independent experiments. (B) Graph showing the median fluorescence intensity (MFI) of CD69 staining of T cells at each time-point. Data are representative of two independent experiments.

BALB/c splenocytes were prepared as follows. Spleens were mechanically disrupted then the tissue forced through cell strainers into RPMI 1640 medium. The cells were purified by density centrifugation (Histopaque-1083; Sigma-Aldrich).

**Figure 5:**
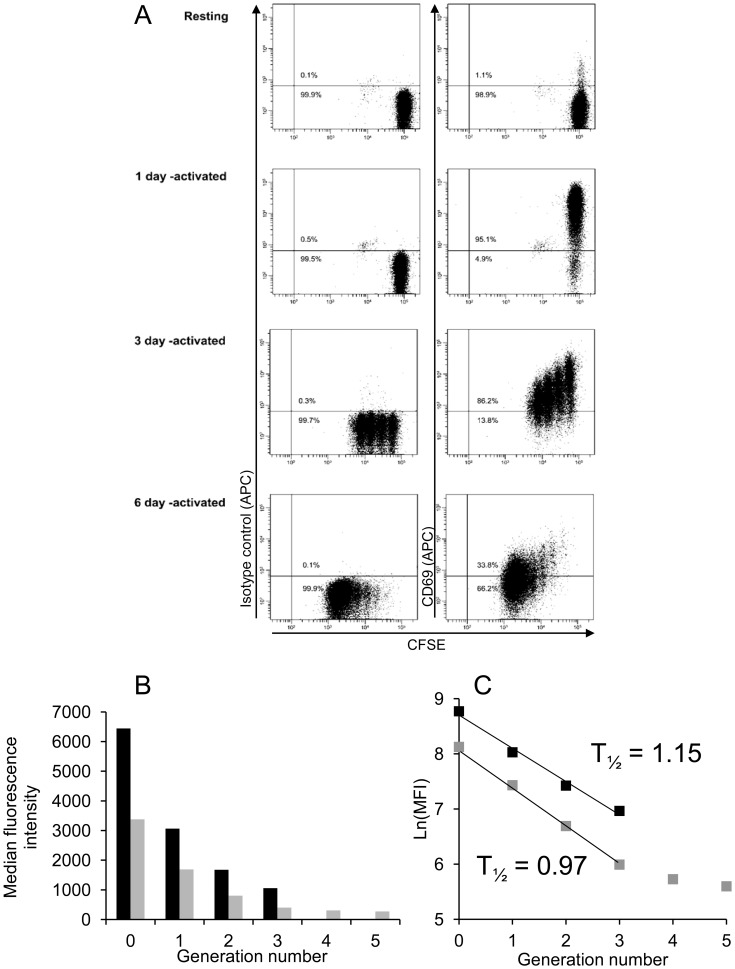
Analysis of CD69 expression following T cell mitosis. (A) Two-parameter dot plots showing variation in CD69 expression with dilution of CFSE during T cell mitosis. Data are representative of two independent experiments. (B) Distinct generations of three-day (black bars) and six-day (grey bars) activated T cells were identified by their CFSE-mediated fluorescence. The MFI of specific CD69 staining of each group of cells was calculated by subtracting the median fluorescence intensity associated with the isotype-matched control antibody from that with the monoclonal antibody against CD69. (C) Natural logarithms of the MFI values were calculated and plotted against the corresponding generation numbers to allow measurement of the generation half-life of CD69 expression. Data are representative of two independent experiments.

### Enzyme-linked Immunosorbent Spot (ELISPOT) Assay

Ninetysix-well format Immobilon MultiScreen-P plates (Millipore; Watford, UK) were coated with IFN-γ capture antibody [clone AN18] (Mabtech; SE-131 28 Nacka Strand, Sweden) diluted into carbonate-bicarbonate buffer (Sigma-Aldrich) overnight at 4°C. These plates were washed twice with PBS and blocked with RPMI 1640 medium supplemented with 10% FBS, 100 U/ml penicillin and 0.1 mg/ml streptomycin (all Sigma-Aldrich) for 1 h at room temperature. Mixed leukocyte reaction assays were then performed in each well by mixing 1×10^4^ C57BL/6 popliteal lymph node-derived cells with 2×10^5^ BALB/c splenocytes in a total volume of 200 µl RPMI 1640 culture medium containing 50 µM 2-mercaptoethanol (Sigma-Aldrich). After incubation for 18 h at 37°C the cells were discarded and the plates washed six times with PBS. A biotinylated IFN-γ detection antibody [clone R4–6A2] (Mabtech), diluted in PBS, was applied overnight at 4°C. The plates were washed six times with PBS. The streptavidin-alkaline phosphatase conjugate (Mabtech) was diluted in PBS and applied for 1 h at room temperature. The plates were washed another six times with PBS and 50 µl of BCIP/NBT liquid substrate system for membranes (Sigma-Aldrich) was added to each well. After development for 5 min at room temperature, the reaction was stopped by removal of substrate and washing with tap-water. Developed spots were enumerated using an ELISPOT reader (AiD; Strassberg, Germany).

**Figure 6:**
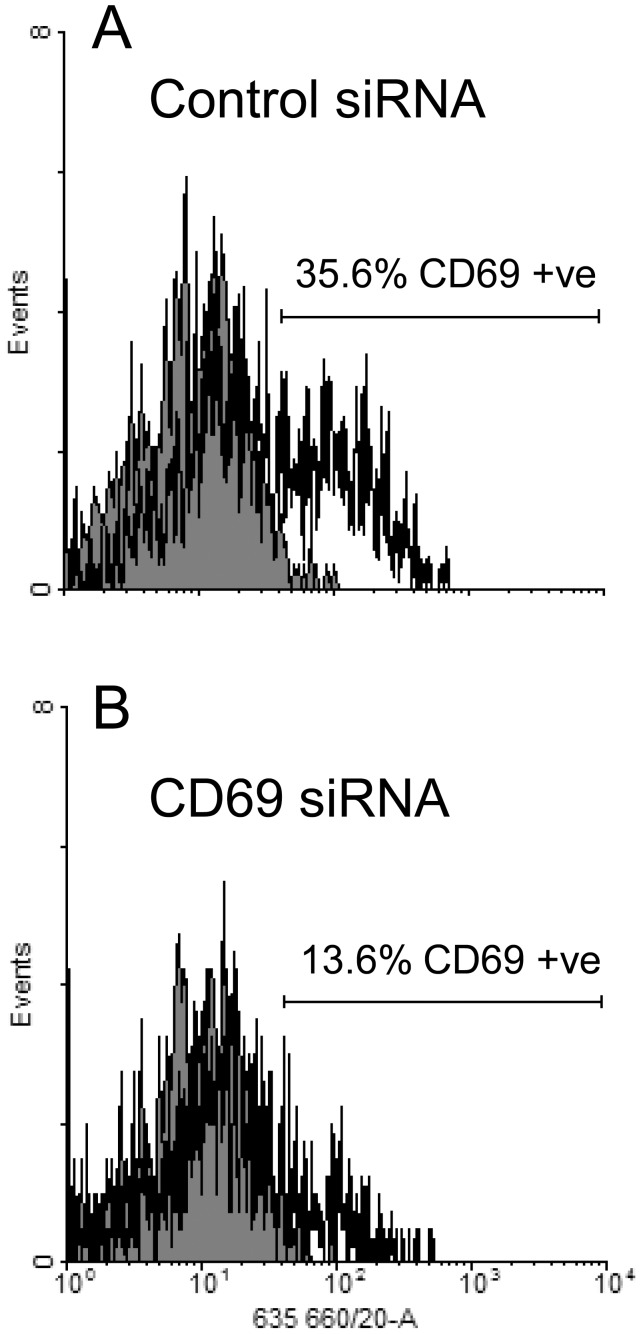
Expression of CD69 expression by activated T cells transfected with siRNA. Resting T cells were transfected with either CD69-specific siRNA or negative control siRNA then stimulated with CD3/CD28 beads for 24 hours. Samples from each cell population were taken, stained for CD69 (open histogram) or with an isotype control antibody (grey histogram) and analysed by flow cytometry.

### Cell Isolation, Culture and Activation

Total CD3^+^ T cells were derived from human peripheral blood or platelet-depleted leukocyte cones (NHS Blood and Transplant Service, UK). They were isolated using an erythrocyte-rosetting negative-selection kit (StemCell Technologies; Grenoble, France) and cell separation by centrifugation across Lympholyte H (Cedarlane Laboratories; Ontario, Canada). T cells were cultured in X-VIVO 15 medium (Lonza; Slough, UK) and activated with Dynabeads Human T Activator CD3/CD28 (Life Technologies; Paisley, UK), at a ratio of one bead per cell and a starting culture density of 1×10^6^ cells per ml.

**Figure 7:**
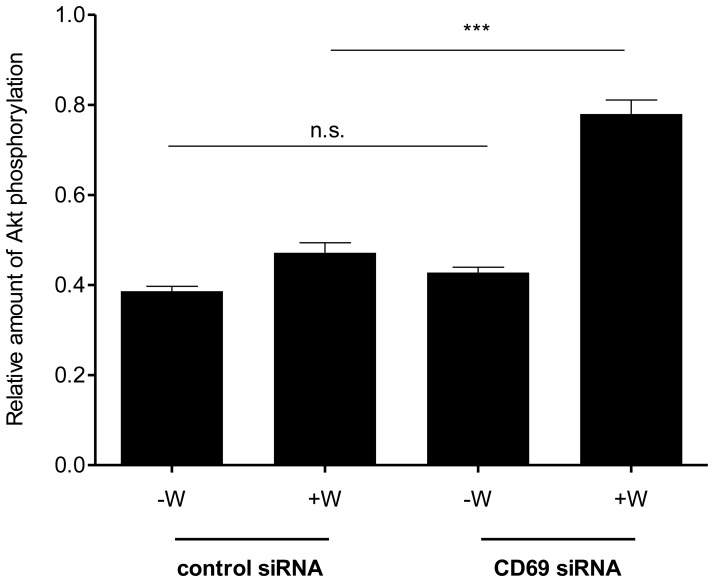
Measurement of Akt phosphorylation following stimulation of siRNA- transfected T cells with SEW2871. Equal numbers of the siRNA-transfected, activated T cells were treated with either 10 µM SEW2871 (+W) or vehicle (-W) for 10 minutes and both pAkt (serine 473) and total Akt were detected using a cell-based ELISA. The relative amounts of pAkt in the samples were calculated by normalising the fluorescent signal value corresponding to pAkt to that of total Akt in each experimental well. Graph shows means ± s.e.m. of triplicate determinations. Data are representative of three independent experiments.

### Antibodies, Cell Labelling and Flow Cytometry

For analysis of mouse cells, the antibodies were CD3 [clone KT3] (AbD Serotec) and CXCR3 [clone 220803] (R&D Systems). The antibodies used for human cells were S1PR1 [clone 218713] (R&D Systems) and CD69 [clone FN50] (BD Biosciences). In some cases T cells were labelled by incubation with 1 µM CFSE-DA (carboxyfluorescein diacetate N-succinimidyl ester; Sigma-Aldrich) for 5 min at 37°C in PBS +0.1% (v/v) FBS. They were then chilled rapidly on ice and washed with cold RPMI 1640 before use. Data was acquired on a FACSCanto II instrument (BD Biosciences) and analysis performed using FACSDiva 6.1.3 (BD Biosciences) and FlowJo 7.6 (Treestar; Ashland, Oregon, USA) software.

### Real Time PCR

RNA was prepared from cell pellets using TRI Reagent (Sigma-Aldrich). cDNA was synthesised from approximately 1 µg RNA per sample using SuperScript III Reverse Transcriptase and random hexamers as primers (Life Technologies). Expression of S1PR1 was determined semi-quantitatively using a specific TaqMan Gene Expression assay (Hs00173499_m1), with 18S ribosomal RNA as the reference (Hs99999901_s1; both Applied Biosystems, Life Technologies; Paisley, UK). The amplifications were run on an ABI Prism 7000 instrument (Applied Biosystems).

### Protein Assays

T cells were lysed in a buffer containing CelLytic M (Sigma-Aldrich) + Mini Complete Protease Inhibitor cocktail (Roche; Welwyn, UK) + Halt Phosphatase Inhibitor cocktail (Thermo Fisher Scientific; Loughborough, UK). The concentration of protein in cell lysates was determined by BCA assay (Thermo Fisher Scientific). Equal masses of protein (routinely 10–50 µg) were loaded into each lane of a single gel and subjected to 12% resolving SDS-PAGE. Transfer to polyvinylidene difluoride membrane (GE Healthcare; Little Chalfont, UK) was by wet electroblotting. Membranes were blocked with 5% immunoglobulin-depleted BSA (Sigma-Aldrich) for 1 h before immunoblotting. The primary antibodies used were: phospho(S473)-Akt [clone D9E] and pan Akt [clone C68E7] (both New England Biolabs; Hitchin, UK). The secondary antibody was horseradish peroxidase-conjugated anti-rabbit IgG (Sigma-Aldrich).

Phospho(S473)-Akt and total Akt were also detected using a cell-based ELISA (R&D Systems). Approximately 10 000 T cells were added to each well of a 96-well format plate (previously coated with 10 µg/ml poly-lysine (Sigma-Aldrich) for 30 min) and left to attach for 1 h. The cells were treated for the desired length of time then fixed with 8% formaldehyde. Phospho(S473)-Akt and total Akt were detected by double immunoenzymatic labelling. The relative quantities of the proteins were deduced from the intensities of spectrally distinct fluorescences associated with each target. The detector was a Dynex MFX instrument (Worthing, UK) operating with excitation and emission wavelengths of 540 nm and 600 nm for phospho(S473)-Akt, and 360 nm and 450 nm for Akt.

### Gene Knockdown

Resting T cells were transfected with 100 pmol siRNA per million cells by electroporation (Nucleofector I instrument; Lonza). For CD69 knockdown, an equimolar mixture of three 21 nucleotide duplexes (MISSION siRNA; Sigma-Aldrich) was used. The single strand sequences were:

5′ GAGUUAGAUGUUGGUACUA 3′

5′ CUACUCUUGCUGUCAUUGA 3′

5′ CUCUCAUUGCCUUAUCAGU 3′

Separately, as a control, cells were transfected with an equal mass of MISSION siRNA Universal Negative Control 1 (Sigma-Aldrich).

After transfection, the T cells were rested for about 5 h in X-VIVO 15 before addition of Dynabeads Human T Activator CD3/CD28 at a ratio of one bead per cell. 24 hours later the cells were analysed directly for CD69 expression or viable cells were sorted using a FACS Aria II (BD Biosciences) instrument for phospho-protein analysis.

### Statistics

Prism 4.0c (GraphPad Software; La Jolla, California, USA) was used for statistical analyses. Comparisons between groups were made using the Student’s *t*-test for unpaired data. P values ≤0.05 were considered significant.

## Results

### The Egress of Alloactivated T cells from Lymph Nodes is Dependent on Intact S1P Receptor Signalling

An initial series of experiments demonstrated that unilateral foot pad sensitisation of C57BL/6 mice with BALB/c splenocytes produced a difference (P<0.01) in popliteal node cellularity after 6 days, with a mean of 2.01×10^6^ cells (s.e.m. 0.17×10^6^) recovered from the sensitised side and 4.88×10^5^ (s.e.m. 2.92×10^5^) from the uninvolved contralateral node (data not shown). Efficacy of the drug FTY720-P in our animal model was tested by measuring the effect of its administration on the frequency of peripheral blood CD3^+^ cells. Daily dosing over two days resulted in a greater than 90% depletion of CD3^+^ cells from peripheral blood (Fig 1A and B). A second series of experiments showed that the overall yield of cells from the reactive popliteal node of sensitised animals was not changed from control values by daily treatment with FTY720-P (P>0.05; Fig 1C).

Measurement by ELISPOT of the frequency of IFN-γ producing, alloreactive T cells in the population recovered from reactive popliteal lymph nodes showed a difference (Fig 1D; P<0.01) between animals treated with the drug vehicle and FTY720-P, with a mean 1.9-fold enrichment of these cells in nodes from the drug treated animals. Analysis of the expression of the T cell activation-associated chemokine receptor CXCR3 in the popliteal node-derived cell population (Fig 1E) also showed a mean 1.9-fold enrichment of these cells in drug-treated animals (Fig 1F; P<0.05).

### T cell Activation is Associated with a Transient Loss in S1PR1 Signalling Capacity

Longitudinal analysis of the expression of S1PR1 gene following T cell activation by stimulation of CD3 and CD28 (Fig 2) showed a marked reduction in transcription after 24 h; a similarly reduced level of mRNA encoding this receptor was observed 3 days after T cell activation.

To test for the presence of functional S1PR1 at the cell surface, T cells were stimulated with the specific agonist SEW2871 and activation of the downstream signalling component Akt measured. When resting cells were stimulated with the specific agonist SEW2871 there was rapid formation of the phosphoserine (473) derivative of Akt (pAkt) (Fig 3A). Stimulation of T cells with this agonist 24 h after the activation of T cells did not increase the level of pAkt. However, an increased level of pAkt was observed when T cells were stimulated with SEW2871 on either day 3 or 6 after mitogenic activation. Additionally, Cell-based ELISA was used to quantify the relative amounts of pAkt and Akt in each cell population before and after cell stimulation with SEW2871. Data from three independent donors (Fig 3B) show that the transient reduction in S1PR1-mediated intracellular signalling was only apparent 1 day after T cell activation.

### Analysis of CD69 Expression Following T cell Activation

Flow cytometric analysis of human T cell surface expression of CD69 showed that almost no resting cells expressed this antigen (Fig 4A). However, almost all these cells expressed CD69 when analysed 1 and 3 days after mitogenic T cell activation (Fig 4A). Although the T cells analysed on days 1 and 3 after activation were uniformly positive for CD69 expression, quantification of the median level of antigen expression per cell demonstrated a marked reduction between days 1 and 3 after T cell activation (Fig 4B).

The rate of loss of CD69 from the surface of activated T cells was analysed by fractionating the dividing cells on the basis of CFSE dilution (Fig 5A). This experiment showed that none of the cells had divided on day 1, maintaining maximum CD69 expression. However, the overall level of CD69 expression observed on day 3, for example, was the sum of separate levels expressed by T cell subpopulations which had divided 0, 1, 2 or 3 times (Fig 5B). On both days 3 and 6 after activation, analysis of the rate of decrease of CD69 expression between cell cycles 0 and 3 showed a similar half-life of 1.15 and 0.97 mitotic division cycles respectively (Fig 5C).

### Loss of T cell Expression of CD69 Correlates with Increased S1PR1 Signalling

A series of experiments was performed to demonstrate the potential to reduce CD69 expression by transfection of resting human T cells with sequence-specific siRNA sequences prior to mitogenic activation. After gating to exclude the T cells damaged during transfection, the percentage of cells expressing CD69 1 day after mitogenic activation (Fig 6) was reduced from 35.6% (control siRNA) to 13.6% (CD69-specific siRNA); the corresponding median fluorescence values were 24.1 and 11.4 respectively.

A series of 3 separate experiments was then performed in which viable and non-viable T cells were separated after transfection by fluorescence-activated cell sorting. These cells were then activated by stimulation of CD3 and CD28 for 1 day and the pAkt/total Akt ratio was quantified after stimulation with the specific S1PR1 agonist SEW2871. This study demonstrated that treatment with the CD69-specific siRNA sequence markedly increased the pAkt signal generated by stimulation of S1PR1 (P<0.001; Fig 7).

## Discussion

The current model for regulation of the egress of T cells from lymphoid tissue has two facets: the situation in normal circumstances and that during non-specific lymph node inflammation. The current study extends this by using *in vivo* and *in vitro* methods to examine how the exit of antigen-activated T cells from lymph nodes is controlled.

Published work has shown that the S1P agonist FTY720 can block the routine efflux of lymphocytes from lymphoid tissue during homeostasis. However, there has been some debate as to the extent to which the efflux of activated T cells from lymph nodes is S1P-dependent. Habicht *et al.* adoptively transferred TCR-transgenic, allospecific T cells along with an allograft and showed that effector memory T cells are trapped in regional lymphoid tissues by FTY720 treatment [24]. It has been known for some time that whereas FTY720 treatment efficiently depletes naïve T cells from peripheral blood, antigen-experienced cells are not affected [25]. Furthermore, using an *in vivo* model of contact hypersensitivity, Nakashima *et al.* showed that FTY720 did not significantly suppress the delayed-type hypersensitivity reaction if administered during the afferent phase [26].

An *in vivo* model of alloimmunity was used in the current study to determine whether or not the egress of activated T cells from lymph nodes is dependent on S1P receptor signalling. The functional S1P receptor antagonist FTY720-P was administered two days after sensitisation with allogeneic cells in the foot pad to minimise unwanted effects on immune priming. This treatment did not alter the total number of cells within the draining popliteal node but led to specific accumulation of the subpopulations of alloreactive and CXCR3-expressing T cells, suggesting that the ability of activated T cells to exit the reactive node is S1P-dependent.

Previous work has shown that it is signalling of S1PR1, and not that of other S1P receptors, that overrides lymph node retention signals to allow T cell exit from lymph nodes [1]. Regulation of this receptor following T cell activation was therefore the focus of the study. The observation that S1PR1 gene expression was decreased by mitogenic activation of human T cells is consistent with a previous demonstration that S1PR1 expression is reduced in mouse T cells following TCR activation [27]. Indeed, it is known that stimulation through the TCR suppresses expression of the transcription factor KLF2, which is a positive regulator of S1PR1 transcription [28]. It is interesting that the magnitude of the signalling response to stimulation of S1PR1 on three-day activated T cells was comparable to that of resting cells, even though the gene expression was approximately three-fold lower in the activated cells. Because of the lack of a monoclonal antibody which can detect cell-surface S1PR1, the relative amounts of protein on resting, one day and three day activated cells could not be directly compared. It also ruled out use of techniques such as fluorescence resonance energy transfer (FRET) to study directly protein – protein interactions with S1PR1. Nevertheless, it is possible to infer from our data that control of S1PR1 gene expression has only a minor role in the regulation of receptor-mediated intracellular signalling following T cell activation.

The expression of CD69 on the T cell surface was at a maximal level 24 h after mitogenic activation. At this time the potential for agonist-induced S1PR1 signal transduction to elicit phosphorylation of Akt was completely inhibited. Although most of these T cells still showed positive cell-surface CD69 after 3 days, the median level of CD69 expression was greatly reduced. At this time the cells showed an intracellular signalling response following stimulation with the S1PR1-specific agonist SEW2871 which was similar to that of resting T cells. This is consistent with a report showing that T cells which have been activated *in vivo* re-acquire at least some responsiveness to S1P by day 3 [5].

Cell cycle analysis of changes in the cell-surface expression of CD69 by activated T cells showed that the level of this antigen decreased by almost exactly one half during each of the first 3 cell divisions. This is consistent with there being little neosynthesis of CD69 after the first mitotic division, with the existing protein dividing equally between the daughter T cells. A previous study supports this model by demonstrating the mRNA encoding CD69 is only detectable during the first 24 hours after T cell activation [16]. The current study showed no evidence of T cell proliferation during this period.

The dilution of CD69 protein during T cell mitosis provides a potential control mechanism for S1PR1 signalling and is consistent with an established model [29] in which the division history of an individual T cell is a major determinant of its fate. Those T cells that have a short division history would be least differentiated but, by expressing a high level of CD69 protein, would be inhibited from exiting the lymph node. In contrast, T cells that have divided several times would become more differentiated. These cells would express low levels of CD69 allowing the egress of short-lived effector T cells from the lymph nodes.

The potential to regulate S1PR1 activity directly by reduction of the level of CD69 protein expression was examined by transfection of T cells with specific siRNA sequences prior to mitogenic activation. The act of transfection of T cells with either control or CD69-specific siRNA sequences non-specifically prevented a sub-population of these cells from upregulating CD69 expression following T cell activation. Nevertheless, stimulation of control and experimental T cell transfectants with SEW2871 after mitogenic activation for 24 h revealed a highly significant difference. In common with non-transfected cells, those T cells which had received a control siRNA sequence showed no significant accumulation of pAkt following treatment with the specific S1PR1 agonist. In contrast, T cells which had been transfected with the specific siRNA sequence showed a marked accumulation of pAkt following stimulation with the agonist. These data suggest that the re-acquisition of responsiveness to S1PR1 stimulation observed 3 days after T cell activation is a consequence of the decrease in CD69 expression produced by between 1 and 3 cycles of mitosis.

The existence of a CD69-mediated mechanism to control S1PR1 signalling, and therefore by implication to inhibit normal T cell efflux from lymph nodes, supports the potential for therapeutic reinforcement of this natural process in order to produce the depletion of activated allospecific T cells from the circulation of transplant patients. This provides further impetus for clinical development of novel immunosuppressive agents which inhibit the S1P receptor system.
